# Supplementary material for: Conditioning Factors of Linearized Wood’s Function Lactation Curve Shape Parameters, Milk Yield, Fat and Protein Content in Murciano-Granadina Primiparous Does
Source: Animals (Basel). 2020 Nov 15;10(11):2115. doi: 10.3390/ani10112115 (PMC7697502; doi:10.3390/ani10112115)
Supplement: Supplementary file 1 [file animals-10-02115-s001.pdf]

Table S1: *Number of lactations per farm.*

Farm	Frequency	Farm	Frequency	Farm	Frequency	Farm	Frequency	Farm	Frequency	Farm	Frequency
**AAB**	183	**CP**	88	**GFB**	205	**GPA**	270	**JAC**	136	**OB**	55
**AAD**	3	**CR**	7	**GFG**	147	**GPC**	303	**JB**	33	**OMM**	17
**AAG**	180	**CRV**	30	**GFJ**	1	**GPF**	23	**JBF**	110	**PM**	31
**AAO**	42	**CSN**	340	**GGE**	217	**GPM**	316	**JCS**	190	**PMA**	5
**AAR**	12	**CYR**	102	**GHA**	201	**GPP**	1	**JEG**	32	**PPO**	42
**AAT**	31	**DAD**	442	**GHG**	8	**GPS**	179	**JH**	45	**PR**	28
**ABC**	175	**DMA**	1	**GIL**	192	**GPV**	26	**JL**	22	**RBB**	281
**AC**	166	**DP**	93	**GIO**	657	**GRA**	3	**JLR**	3	**RBG**	84
**ACG**	9	**EBM**	412	**GIS**	116	**GRE**	392	**JRE**	103	**RCH**	6
**ACM**	99	**ECG**	2	**GJC**	91	**GRF**	9	**JRT**	4	**RK**	93
**ADH**	7	**FCR**	38	**GJI**	63	**GRJ**	234	**JSG**	19	**RLM**	16
**AFH**	41	**FD**	4	**GJL**	82	**GRR**	425	**JUN**	72	**RO**	13
**AG**	488	**FH**	1	**GJN**	252	**GRS**	2	**KAH**	36	**RRL**	751
**AGG**	55	**FM**	36	**GJP**	476	**GSJ**	87	**KAJ**	2	**RSR**	11
**AGH**	3	**FMM**	135	**GJR**	112	**GTJ**	58	**KAN**	15	**SAR**	56
**AJ**	55	**FRA**	297	**GLG**	86	**GTN**	258	**KAV**	733	**SB**	69
**AJG**	559	**FYA**	43	**GLH**	194	**GUI**	10	**KH**	25	**VJP**	53
**ALH**	91	**GAE**	63	**GLJ**	202	**GXG**	25	**KY**	178	**WAD**	378
**AM**	50	**GAM**	56	**GLM**	49	**GYR**	67	**KYB**	25	**WAG**	75
**AMC**	233	**GAP**	303	**GLP**	34	**HC**	14	**KZ**	7	**WAJ**	16
**AUG**	161	**GBA**	39	**GLS**	133	**HN**	153	**LPP**	522	**WAM**	34
**AV**	246	**GBB**	93	**GLV**	9	**HP**	10	**LR**	18	**WD**	131
**AVE**	106	**GBM**	176	**GME**	317	**HR**	17	**LRP**	185	**WJ**	55
**AVR**	8	**GCB**	38	**GMF**	35	**HZC**	28	**LW**	108	**XY**	12
**BAB**	98	**GCG**	84	**GMG**	630	**IAD**	260	**MG**	203	**YK**	52
**BJJ**	62	**GCM**	185	**GMH**	225	**ICN**	3	**MGE**	129	**YM**	24
**BS**	4	**GDD**	77	**GMJ**	79	**IE**	19	**MI**	231	**YO**	32
**CF**	13	**GDL**	217	**GND**	132	**IF**	2	**MLA**	65	**ZAF**	99
**CIK**	9	**GDP**	39	**GOM**	347	**IK**	230	**MMA**	155	**ZS**	36
**CL**	342	**GDS**	350	**GOP**	100	**IQ**	264	**MO**	23		
**CLH**	33	**GEA**	142	**GOT**	189	**IR**	51	**MT**	96		
